# An interdisciplinary, family-centered approach to treating pediatric obesity in an 11-year-old female: a case report

**DOI:** 10.1186/1757-1626-2-6677

**Published:** 2009-06-03

**Authors:** Rachel A Keaschuk, Amanda S Newton, Tesia G Kaczmarek, Geoff DC Ball

**Affiliations:** 1 Pediatric Centre for Weight and Health, Stollery Children's HospitalEdmonton, ABCanada; 2 Department of Pediatrics, Faculty of Medicine and Dentistry, University of AlbertaEdmonton, ABCanada

## Abstract

Pediatric obesity has become increasingly prevalent over the past 2-3 decades. Recently-published clinical practice guidelines and expert recommendations provide guidance for obesity treatment, but therapy is often complicated by a host of medical, behavioural, psychosocial, and interpersonal issues. We report the case of an 11-year-old obese girl and her family referred for weight management. Our case underscores the need for an interdisciplinary, family-centered approach to the assessment and treatment of pediatric obesity, and highlights the value of understanding familial complexities that often accompany this health issue. The importance of utilizing multiple health indicators to assess weight management ‘success’ is discussed.

## Introduction

Given the high prevalence of obesity and its concomitant health risks, clinicians have an important therapeutic role to play in helping obese children and their families optimize weight-related cognitions and behaviors. The recent publication of clinical practice guidelines and expert recommendations offer clinicians evidence-based guidance for the treatment of pediatric obesity [1,2]. Building on this evidence, and based on our team's clinical experience, we believe there are two central issues that should guide how these recommendations are implemented. First, there is a critical need for an interdisciplinary team assessment at presentation to determine families' medical, behavioral, psychosocial, and interpersonal issues. Although an interdisciplinary assessment has been encouraged [1,2], the interplay between medical, behavioural, and psychosocial assessments have not been outlined in detail and examples of tools and procedures for use in the clinical setting have not been specified. Second, to identify families' capabilities and readiness to make and maintain positive changes, a thorough exploration and understanding of familial complexities is needed for clinicians to help families achieve weight management success. Framed by these two issues, the case below highlights our team's approach in the context of one family.

## Case presentation

**Figure 1.**
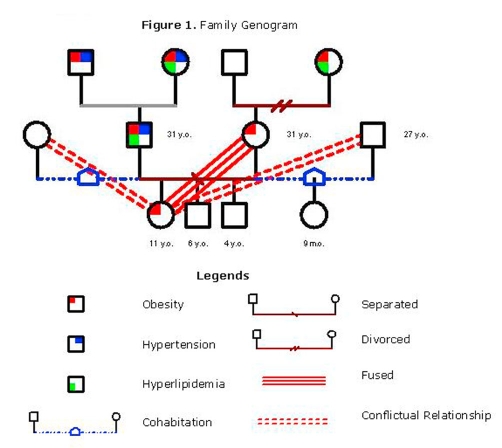
Family Genogram.

An 11-year-old Caucasian (Canadian) obese girl was referred for weight management with her family. Prior to beginning treatment, the family underwent medical, behavioural, and psychosocial assessments by a pediatrician, nurse, dietitian, exercise specialist, and psychologist. The patient's BMI at intake was 29.9 kg/m^2^ (age- and sex-specific BMI >98^th^ percentile). She also had a high waist circumference (>95^th^ percentile). Several obesity-related co-morbidities were identified including mildly elevated blood pressure (>90^th^ percentile), elevated total and LDL cholesterol (75^th^ and 90^th^ percentiles, respectively), and low HDL cholesterol (10^th^ percentile) (Table 1). While fasting glucose was normal, insulin was elevated; acanthosis nigricans, a marker of insulin resistance, was observed at the neck. Family medical history indicated obesity, hyperlipidemia, and hypertension in both maternal and paternal families (Figure 1). A comprehensive lifestyle behavior assessment, which included a 4-day food record, pedometer log, and physical activity record, was completed. Compared to other obese children enrolled in our weight management center [3], vegetable and fruit intake and physical activity were relatively healthy, but records highlighted several opportunities to make healthier choices, especially regarding intake of high sugar/high fat foods and sedentary activity.

**Table 1.** Physical, behavioural and familial changes before and after interdisciplinary weight management

	Pre-Intervention	Post-Intervention	Percent (%) Change
**Traditional Measures:**			
Height (cm)	160.3	162.2	1.2
Weight (kg)	76.3	76.8	0.7
BMI (kg/m^2^)	29.9	29.2	-2.3
BMI Percentile	98.5	97.9	-0.6
Waist Circumference (cm)	103.9	100.2	-3.6
**Extended Measures:**			
Body Fat (%)	40.6	37.7	-7.1
Systolic Blood Pressure (mmHg)	122	96	-21.3
Diastolic Blood Pressure (mmHg)	80	62	-22.5
Total Cholesterol (mmol/L)	4.77	3.76	-21.2
LDL Cholesterol (mmol/L)	3.26	2.68	-17.8
HDL Cholesterol (mmol/L)	0.91	0.77	-15.4
Insulin (μIU)	10.1	8.5	-15.8
Glucose (mmol/L)	4.6	5.0	8.7
Vegetables & Fruit (svg/d)	5.1	5.5	7.8
Foods Classified as ‘Others’ (%)^1^	65	11	-83.1
Screen Time (hr/d)	4.5	1.5	-66.7
Physical Activity (steps/d)	11,574	8,123	-29.8
Cardiorespiratory Fitness (min:sec)^2^	9:15	14:30	56.8
Family Life Stress (percentile)	>99	97	2
Family Communication (percentile)^3^	40	61	50.0
Family Satisfaction (percentile)^3^	25	66	164
Family Cohesion and Flexibility^4^	1.67	2.38	42.5

1 Includes high energy-dense foods that are high in sugar and/or fat and low in nutrients.

2 Time to exhaustion on a treadmill walking test using a modified Balke protocol.

3 Change is positive from ‘low’ to ‘moderate’ score.

4 Change is positive from ‘low-balanced’ to ‘average-balanced’.

The patient's psychosocial history indicated difficulties with social skills including struggles to develop and maintain friendships; she was accused of bullying repeatedly during the past school year. She reported a preoccupation with her body and appearance, poor self-esteem, and being sensitive to social rejection. Data from the *Child Behavior Checklist* [4] indicated that her symptoms were within the ‘clinical range’ for social problems, internalizing problems, and total problems; scores fell in the ‘borderline range’ for anxiety and depression. Scores on the *Parenting Stress Index* (PSI) [5], a measure of situational stressors, indicated that the mother found her daughter's demandingness and mood stressful, and that she failed to meet parental expectations for attractiveness and pleasantness. A family social assessment indicated a history of instability in the past year (Figure 1). The patient's parents had unexpectedly separated and paternal visitation was sporadic. New additions to family structure occurred (i.e., parental separation, new parental partners, birth of sibling), and there were several family relocations. Throughout her parents' relationship, the patient assumed a parentified role in the family and was struggling to adapt to role changes with the addition of new parental figures. The life stress score on the PSI was elevated. Family functioning, as measured by the *Family Adaptability and Cohesion Evaluation Scales* [6], indicated relatively healthy levels of family cohesion and flexibility, but the mother reported dissatisfaction with the family system and the family's communication patterns. Following these assessments, a team case conference, and discussion with the family, all agreed on the family's need and readiness for weight management.

Subsequently, the patient's mother and partner enrolled in a 16-week, group-based weight management intervention. This evidence-based program was consistent with current pediatric obesity treatment recommendations and included working with parents as *agents of change* on behalf of the family. Lifestyle changes were contextualized within the family system, an approach that can be more effective than working with children exclusively or parents and children together [7,8]. The intervention focused on helping the patient's mother and partner to set nutrition, physical activity, communication, and relationship goals that built on family strengths and enhanced areas (identified by the family) that needed improvement. The patient's mother and partner attended 15/16 sessions. During this time, the patient attended six adjuvant individual psychotherapy sessions that focused on coping with difficult peer situations and depressed mood.

At the end of the intervention, the patient's BMI and BMI percentile decreased slightly, but she was still classified as *obese* (Table 1). Improvements in several metabolic risk factors were also noted. Lifestyle behaviour assessments indicated increased vegetable and fruit intake, decreased intake of high sugar/high fat foods, and reduced screen time. While steps/day decreased, the patient improved her time on the treadmill test by >5 minutes indicating an improved level of cardiorespiratory fitness. The patient's depressive symptoms improved and all subscales of the *Child Behavior Checklist* improved to within the ‘normal range’. Her mother reported a more positive view of her daughter and found her behaviour less stressful. While life stress for the family remained the same, family communication, satisfaction, and family cohesion and flexibility all improved following the intervention (Table 1).

## Discussion

Our clinical assessments in this case included an examination of physical, behavioural, psychosocial, and interpersonal issues. This detailed evaluation assisted us, both clinicians and the family, in identifying health improvements over the course of treatment. It is worth noting that the traditional measurements (Table 1) often taken to evaluate pediatric obesity treatment success may foster a pessimistic view of the family's intervention since only modest anthropometric changes were noted. However, in the absence of intensive therapy (i.e., bariatric surgery), it can take an extended period for a patient's weight status to improve substantially. As an initial goal, one of the key recommendations in pediatric weight management is to achieve weight maintenance, followed by weight loss if warranted. A singular focus on weight loss to gauge treatment success may undermine many other healthy family changes; this supports the extensive measurements we completed provided a comprehensive view of the physiological, lifestyle, and family-related changes. These measures also gave us the opportunity to highlight areas of strength within the family to help encourage them to make further lifestyle changes. By emphasizing the family's positive changes (i.e., reductions in blood pressure and cholesterol, increased dietary quality and cardiorespiratory fitness, and improved family dynamics), the family was able to identify meaningful health improvements and was motivated to continue making changes. While the procedures required to collect this information can be labor-intensive for clinicians and time-consuming for families, these data provide relevant information upon which families can establish specific and tailored treatment goals. In addition, families are able to base their goals on objective data that serve as clinically-meaningful benchmarks.

Our interdisciplinary, integrated approach enabled our team to help this family to develop strategies during the intervention for family-wide changes. Parents were encouraged to understand how their busy schedules and family disruptions impacted lifestyle behaviour goals, and how they could work together as a parenting unit to make changes while being mindful of potential roadblocks. Although this family was attempting to manage many stressors, they also had several strengths. The mother and her partner were highly motivated and were willing to work together to make lifestyle changes for the family rather than just helping their daughter to make changes on her own. The contextual information we derived from clinical interviews with the family, combined with valid and reliable psychosocial and family-related questionnaires [4-6], enabled us to form a comprehensive perspective of this family. This case demonstrates that lifestyle changes are made within the context of family functioning, and that clinicians must be mindful of family complexity, schedules, and norms when making recommendations. For example, if immediate lifestyle behavioural changes prove to be particularly challenging and the psychosocial / familial assessment determines a family is under a high level of stress or dealing with tumultuous personal problems, modest treatment goals or deferral of treatment should be discussed with families. Family complexity does not necessarily limit a family's ability to make changes. If a family has a high level of awareness and has the ability to manage their stressors, treatment should be initiated.

## Conclusion

Lifestyle changes occur for obese children within the context of their families. An integrated interdisciplinary assessment of the family environment and functioning, one that augments traditional weight management metrics, is necessary to make evidence-based (and practical) recommendations. The degree of family complexity, along with their strengths and barriers to change, must also be taken into account. It is necessary to assess a number of health indicators that are relevant to pediatric obesity, not only to identify co-morbid conditions, but to identify areas of improvement as a way of optimizing family motivation. Often, once improvements are made in psychosocial or interpersonal domains, new opportunities emerge for families to make healthier nutrition and physical activity choices. We submit that in lieu of a traditional, reductionistic approach that focuses exclusively on weight loss as the indicator of success, pediatric obesity should be conceptualized as a complex issue that requires an interdisciplinary, integrated, and evidence-based response that includes weight loss as one of several important treatment outcomes. We believe delivering obesity treatment services in this manner will also be beneficial for clinicians who often experience frustration and feel ineffectual when traditional indicators of weight management success are not achieved.

## Abbreviations

None
